# The What Is It?

**Published:** 1881-02

**Authors:** C. Pearson

**Affiliations:** Washington, D. C.


### THE WHAT IS IT.

BY C. PEARSON, M.D., WASHINGTON, D.C.

THE following is a partial copy of a circular recently sent out by a druggist of this city. He will probably be very grateful for having it noticed in THE HOMŒOPATHIC PHYSICIAN, for should this happen to fall into the hands of some of the hybrid school, they will see at once where they can procure their medicines without patronizing a house that persists in styling itself homœopathic. Thus, the circuler reads:

"Thorough Triturations,
Elegant,
Accurate,
Reliable."

"*The value of thorough trituration is so well established that argument is superfluous.*

"The demand for diluted potent medicines, and the impossibility of making them satisfactorily upon extemporaneous prescriptions has induced the subscriber to offer the medical faculty a class of powders, diluted with pure sugar of milk, so that

*ten* grains will contain *one* grain of the medicine; thus affording physicians an opportunity to obtain with accuracy, a minute quantity of the extract or alkaloid desired: finely divided by *thorough trituration.*"

Then follow the names of the drugs, and the dose, among which are the following:

| | | | | |
|---|---|---|---|---|
| "Atropia, | Lactose, | Dose, | one-tenth to one-fifth | grains." |
| "Cinchonidia, | " | " | five or more | " " |
| "Digitalin, | " | " | one-tenth to one-fourth | " " |
| "Morphia, | " | " | one to five | " " |
| "Quinia, | " | " | five or more | " " |

Now here is an "eternal fitness of things." But shades of Galen! only think, one-half grain of quinine or cinchonidia at a dose! Why it would not be a smell for an eclectic homœopath on the Wabash! One of that class would give from fifteen to twenty-four grains of crude quinine at a dose without a *shake!* See *Medical Investigator* for 1879, page 243, also *Medical Advance*, 1878, page 248. Now this is equal to one hundred and fifty to two hundred and forty grains, or from one-forth to one-half ounce of this druggist's trituration at a dose! If he can only succeed in getting the trade of these men, and I hope he will, as I like to encourage home enterprise, they will clean out his whole establishment in a short time. But I fear his preparations will not suit their heroic tastes. They might urge as an objection the inconvenience of feeding to their patients two hundred and forty grains of this powder every two or three hours, or the patients themselves might not take to it kindly. Yet Dr. Smythe, in his work on "Medical Heresies," has shown they do not hesitate to give ten times more medicine than the boldest allopath would dare do; hence, taking the doses recommended by this druggist as the standard, there is no trouble at all in doing this. Witness the following: "fiifteen to thirty grains of chloral and bleeding for puerperal convulsions." (*Med. Invest.*, Nov. 15th, 1880). Aconite 2d; Hamamelis 2d; also a tonic; two haematic blood pills during the twenty-four hours; a clister of one ounce of *Petrocerate*: fiiteen drops of *Hydrastin;* ten drops of Hamamelis, and ten drops of Bell. thoroughly mixed and warmed. (*Med. Invest.*, Sept. 15th, p. 256). *Gelsem.*, fluid extract, five grains, alternately with Aconite, three grains every ten minutes, and Chloroform by inhalation. Next day *Verat. vir.*, ten grains in two ounces of water, alternately with fifteen grains *Nux vom.* in two ounces of water, a teaspoonful every two hours. *slippery elm* poultice over the lungs, Chloroform as often as required (!), mustard poultice to the wrists and feet, extract of *Bell.* to the spine. *Morphia* full dose, Chloroform to be repeated if neces sary (!). Next day not so well; *Acon.* 1st, *Morphine*, *Chloroform*, *Asafœtida* and *Valerian*, each one drachm. *Bell.* to the spine, mustard to the feet and wrists. *Lobelia* and *Ipecac.* each two drachms every fifteen minutes, *Chloral Hydrate* fifteen grains.

Next day not so well. More *Morphine*, *Chloroform* and *Ether*, *Lobelia* and *Ipecac*, two drachms every ten minutes, also a poultice of tobacco, (something new) and fifteen grains of Chloral.

Next day *Elixer Valerianate* of *Ammonia*, and *Quiniae* a teaspoonful every two hours, more *Chloral* and *Chloroform.* (*Med. Invest.*, Oct. 1st, 1880, p. 292.) And yet these men ask us to share with them the responsibility of such treatment as this, and wonder why we organize a separate medical association, or have the temerity to publish a journal wherein such herecies will not be tolerated! The reason is very apparent; we prefer to obey the command of the prophet of old. "Come out of her my people that you partake not of her sins and receive not her plagues." It is not, as they charge, that we are illiberal, or that we have left them. It is because *they* have left homœopathy and us; because we *know* there is a safer and better way, and because we can not and will not be responsible for the failures that have always and will always attend such heterogeneous prescribing. They tell us we would not resort to these crude drugs to save the lives of our patients. This is not true. It is because we know that safety does not lie in this direction. A boy once in climbing a high precipice, looked down, and became frightened and dizzy. His father called to him to look up; he did look up, and was saved. Now why do these eclectics, when there is danger, always look down, when by looking up they might save their patients? Does this display liberality, and a disposition to resort to anything to save life? Their patients gain nothing by such treatment, while they lose the confidence of the community and their own self-respect. Their methods being mistaken for homœopathy, the latter is denounced as a fraud and a failure. Thus hundreds of patients are deterred from resorting to such treatment believing, and justly, too, that it is no better than that of the old school. The people want something better. They care nothing for names or theories, but they want to be cured. They are tired of swallowing drugs, and if they change physicians it is mainly in order to change the treatment. But when quinine, morphine, Chloral, *etc.*, meet them on every hand, what encouragement have they to change? A lady recently called to consult me, saying she had taken medicine constantly for one year from three different homœopathic (?) physicians without experiencing the slightest relief; and it was only the persistent urging of a friend that induced her to call. I gave one powder of the C.M. of *Cal. Carb.*, and in three weeks she returned to tell me she was well. Scores of such incidents could be reported, and hundreds more would be, if it were not for the reasons already given. But *excelsior!* is the word, and legitimate homœoprthy "holds the fort," and will continue to hold it till the "crack of doom," in spite of the allopathic, or the "rational school of Homœopathy." (See *Buffalo Investigator* for Nov. 1880, p. 251.)

